# Life-long consumption of high level of fruits and vegetables reduces tumor incidence and extends median lifespan in mice

**DOI:** 10.3389/fnut.2023.1286792

**Published:** 2023-12-06

**Authors:** Weimin Guo, Edwin F. Ortega, Dayong Wu, Lijun Li, Roderick T. Bronson, Sarah K. Boehm, Simin Nikbin Meydani

**Affiliations:** ^1^Nutritional Immunology Laboratory, JM USDA Human Nutrition Research Center on Aging at Tufts University, Boston, MA, United States; ^2^Rodent Histopathology Core, Harvard Medical School, Boston, MA, United States

**Keywords:** fruits and vegetables, western-style high-fat diet, obesity, aging, tumor incidence, mortality, inflammation

## Abstract

**Objective:**

Epidemiological studies suggest that consumption of fruits and vegetables (FV) is negatively associated with the incidence of certain cancers and mortality. However, a causal relationship has not been demonstrated. Thus, we investigated the effect of life-long consumption of high level of FV on median lifespan, key biological functions, and pathologies in mice fed low-fat (LF) or high-fat (HF) diets and the underlying mechanisms.

**Methods:**

Using a 2 × 2 factorial design, 5 weeks-old male C57BL/6J mice were randomly assigned to one of four groups (*n* = 60/group): LF (LF-C, 10% kcal fat), HF (HF-C, 45% kcal fat) or each supplemented with 15% (w/w) of a unique FV mixture (LF + FV and HF + FV, respectively). Mice were euthanized when one group reached 50% mortality. Body weight and composition, tumor incidence, and death were monitored. Blood levels of lipids and pro-inflammatory cytokines were assessed.

**Results:**

After 21 months of feeding, HF-C group reached 50% mortality, at which time mice in all groups were terminated. HF-C had higher mortality (50.0%) compared to the LF-C group (18.3%, *p* = 0.0008). Notably, HF-FV had lower mortality (23.3%) compared to HF-C group (*p* = 0.008); there was no significant difference in mortality between HF-FV and LF-C groups. Tumors were found in all groups, and were predominantly present in the liver, followed by those of lung, intestine, and seminal vesicle. Tumor incidence in the HF-C group (73.3%) was higher than that in LF-C group (30.0%, *p* < 0.0001). HF + FV group had 23.3% lower tumor incidence compared to the HF-C group (*p* = 0.014). No significant difference in tumor incidence between the LF-C and LF + FV groups was observed. Long-term FV supplementation reduced systemic inflammation and blood lipids.

**Conclusion:**

We provide the first causal evidence that life-long intake of a diet, containing a high level and large variety of FV, decreases tumor incidence and extends median lifespan in mice fed a western-style high-fat diet. These effects of FV are at least in part due to reduced blood levels of pro-inflammatory cytokines and improved dyslipidemia.

## 1 Introduction

Aging is associated with increased risk of cancer incidence (1, 2). Dysregulated immune function and chronic inflammation (“inflammaging”) are key players in susceptibility to age-related diseases such as cancer (3–5). Epidemiologic studies have associated fruits and vegetables (FV) intake with reduced risk of age-related diseases and mortality, as well as increased longevity (6–10), which may, in part be mediated through a reduction in blood pro-inflammatory cytokines and lipids (11–14). Particularly, FV consumption was shown to be negatively associated with cancer risk (15–17). However, a causal relationship between high FV consumption, and age-associated diseases and longevity have not been demonstrated. Recently, we demonstrated, through two short-term prospective studies, that high consumption of a variety of FV, commonly consumed by Americans, causally reduced non-alcoholic fatty liver disease in C57BL6 mice and atherosclerotic plaque formation in LDL-KO mice (18, 19). Briefly, C57BL6 mice were fed a normal-fat, or a high-fat diet supplemented with different amounts of a mixture of FV (0, 5, 10 and 15%, w/w, approximately equivalent to 0, 3–4, 5–7, and 7–9 servings of FV/d for humans) for 20 weeks. The results showed that supplementation with the 15% FV mixture was most effective in reducing high fat diet-induced hepatic steatosis, a risk factor for liver cancer. Further, we showed that these effects were associated with reduced inflammatory cytokine levels (19). Similar effects were observed when mice fed a normal fat diet were supplemented with 15% FV, albeit to a lesser degree (19). Using LDL-KO mice, we further showed that supplementation of a high-fat, high cholesterol diet with 15% FV mixture significantly reduced liver steatosis and atherosclerosis plaque formation (20). These effects were associated with reduction in pro-inflammatory cytokines and blood lipids. Reduction in inflammatory markers and oxidative stress may be an underlying mechanism for the purported FV-induced effects on health (21).

While these studies suggest that a high FV intake may attenuate age-related diseases leading to healthier and longer life span, to date, no prospective, long-term studies have been conducted to demonstrate that life-long consumption of higher level of FV reduces age-associate pathologies or extends median life span. Thus, the objective of the current study was to determine the effect of long-term supplementation with a 15% FV mixture (the optimal level determined from previous studies) (19, 20) in the context of a low fat or a high fat diet, on age-associated pathologies and median life span. As initial steps to investigate the underlying mechanisms, we determined diet-induced temporal changes in blood lipids and pro-inflammatory markers. These markers were selected based on a plethora of evidence that demonstrates their role in the aging process and age-associated diseases as well as their potential to be influenced by FV intervention (22, 23).

## 2 Materials and methods

### 2.1 Animals and diets

Four-weeks-old male C57BL/6J mice were purchased from the Jackson Laboratory (Bar Harbor, ME, United States) and housed at the animal care facility of the Jean Mayer USDA Human Nutrition Research Center on Aging at Tufts University. Mice were individually housed and received diet and water *ad libitum*. The low-fat control (LF-C, 10% kcal fat) and high-fat control (HF-C, 45% kcal fat) diets were purchased from Bio-Serv, Inc. (Flemington, NJ, United States), and the fruit and vegetable (FV) powder (patent pending) was provided by VDF FutureCeuticals, Inc. (Momence, IL). The details of FV composition were reported previously (19). Briefly, the mixture was selected based on 24 fruits and vegetables commonly consumed by Americans at the percentages determined by the average consumed (USDA census data, average *per capita* daily consumption from 2003 to 2008) (24). The protocols for animal experiments were approved by the Institutional Animal Care and Use Committee of Tufts University. Briefly, after 1 week of acclimation, during which time all mice were fed LF-C diet, mice were randomly (weight-matched) assigned to one of four diet groups (*n* = 60/group) and fed LF-C, HF-C, or each diet supplemented with 15% (w/w) of the FV powder (equivalent to 7–9 servings FV/d for human) (LF + FV and HF + FV, respectively). All mice were kept under the same condition, and individually housed in the same room. The only variable was the type of diet. The mice were continuously fed the four different types of diets every day starting from 5 weeks of age until the end of the study. The nutrient composition of experimental diets is provided in Table 1.

**Table 1:** Nutrient composition of experimental diets.

Diet group	F&V content (% w/w)	Protein (kcal %)	Carb (kcal %)	Fat (kcal %)	Energy density (kcal/g)	Total fiber (μg/kcal)	Soluble fiber (μg/kcal)	Insoluble fiber (μg/kcal)
LF-C	0	20	70	10	3.82	0.0123	0	0.0123
LF + FV	15	18.8	72.2	9.1	3.73	0.1080	0.0338	0.0742
HF-C	0	20	35	45	4.70	0.0123	0	0.0123
HF + FV	15	18.9	41	40.3	4.56	0.1080	0.0338	0.0742

Body weight, food intake, body composition, blood cytokine levels, and blood lipid profile were monitored longitudinally at different time points. Blood was collected for plasma or serum isolation at 12 and 21 months, respectively, and stored at −80°C for further analysis. We had intended to collect blood at 6 months as well but were not able to do that due to COVID-19 restrictions. Mice were observed for health status throughout the study and moribund mice were euthanized and preserved in formalin jars. Death was recorded throughout the study. All mice were euthanized with CO_2_ followed by exsanguination when the first group reached 50% mortality.

### 2.2 Weight and body composition analysis

Mice were weighed weekly. Body composition (% fat and lean tissue) was assessed by using rodent magnetic resonance imaging system (Whole Body Composition Analyzer, EchoMRI, Houston, TX) after 6 and 16 months of dietary intervention.

### 2.3 Measurement of blood lipidomic profile

Lipidomic profiles of plasma (12 months samples) and serum (21 months samples) were analyzed using LC-MS/MS (AB SCIEX 4000 QTRAP) as previously reported (25) by Virginia Commonwealth University Massey Cancer Center Lipidomics Shared Resource. To compare changes in lipid profiles at 12 vs. 21 months samples, we applied a normalization/correction factor to the plasma samples. To determine the correction factor, blood was collected from 4 control mice. Each control blood sample was divided into two portions, one was used to isolate plasma and the other to isolate serum. The 4 pairs of control plasma and serum samples were analyzed for lipidomic profile concomitantly with 12 months plasma samples and 21 months serum samples to determine the correction factor.

### 2.4 Measurement of blood lipid profile and blood pro-inflammatory cytokine levels

Serum or plasma pro-inflammatory cytokine levels were determined using the MSD V-Plex Proinflammatory Panel I Mouse Kit (Meso Scale Discovery, Gaithersburg, MD, United States). Serum lipid profile including total cholesterol (TC), triglyceride (TG), and HDL-c were measured as previously reported (19). Serum levels of LDL-c and VLDL-c were calculated by Friedewald–Fredrickson’s equation (26). Similar to the blood lipidomic profile analysis, the 4 pairs of control plasma and serum samples were analyzed for blood lipid profile and blood pro-inflammatory cytokine levels, and a correction factor was applied to the resulting lipid profiles and cytokine levels.

### 2.5 Statistical analysis

All results were tested for normality and homogeneity of variance. Survival analysis was performed using the Kaplan–Meier (log-rank) test. The two-sided Fisher’s exact test was used for the comparison of tumor incidence. Two-tailed student’s *t*-test (for unpaired samples) and two-tailed paired *t*-test (for paired samples) were used for the comparison of difference in means between two groups. For comparisons of more than two groups, one-way ANOVA and *post hoc* Tukey’s test were used to assess the differences among different diet groups, and two-way ANOVA was used to assess overall effects of age, treatment, and treatment × age interactions. Spearman’s correlation coefficients were calculated to determine correlations among the clinical and biochemical variables. Data analysis was conducted using SPSS (version 28.0.0.0) (IBM Corporation, Armonk, NY, United States) and graphics were generated using GraphPad Prism (version 9.4.0) (GraphPad Software, Inc., La Jolla, CA, United States). Data are presented as means ± SEM. Differences at *p* < 0.05 were considered significant.

## 3 Results

### 3.1 Dietary FV supplementation prolonged lifespan in mice

**Figure 1:**
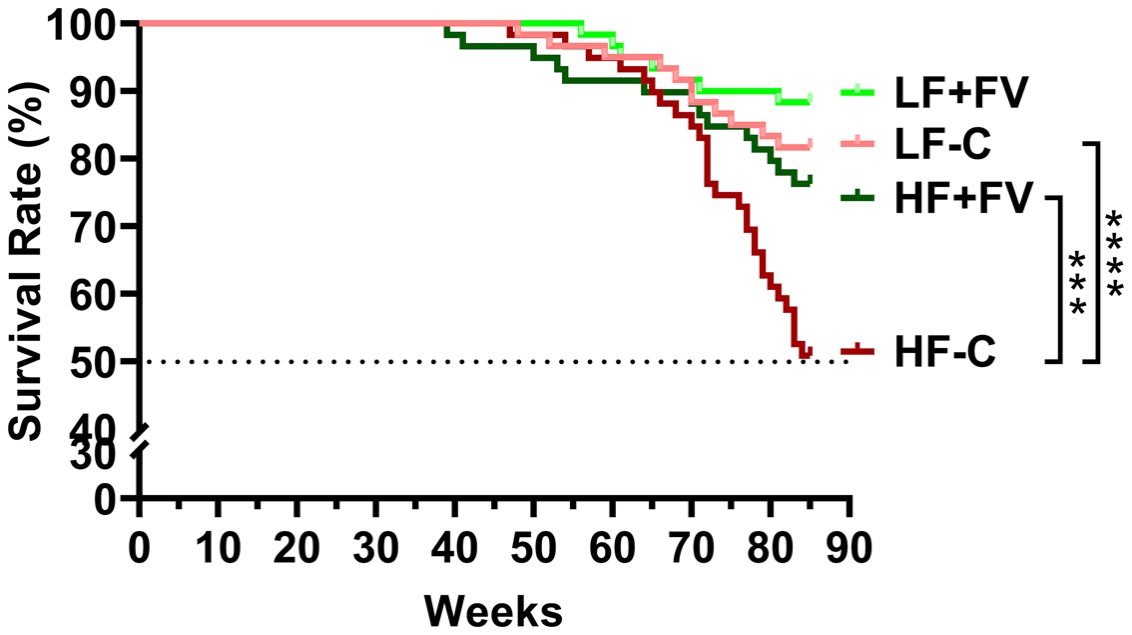
FV supplementation increases median life span. Mice (60/group) were fed LF-C (10% fat), HF-C (45% fat), or each diet supplemented with 15% FV (LF + FV, HF + FV) from when they were 5 weeks old until the first group reached 50% mortality, which was the HF-C group at 21 months. Survival analysis was performed using the Kaplan–Meier (log rank) test. ^***^p < 0.01 and ^****^p < 0.001.

After 21 months of feeding the respective diets, the HF-C group was the first to reach 50% mortality. Kaplan–Meier survival curve analysis demonstrated that at termination, the HF-C group had higher mortality (50.0%) compared to the LF-C group (18.3%, *p* = 0.0008). Notably, the HF + FV group had significantly lower mortality (23.3%) compared to the HF-C group (*p* = 0.008); there was no significant difference in mortality between the HF + FV group and the LF-C group. Mortality in LF + FV group was slightly lower (11.7%) than that in the LF-C group (18.3%), but the difference did not reach statistical significance (Figure 1). Our results demonstrate that dietary FV supplementation significantly prolonged median lifespan in mice fed a high-fat western-style diet.

### 3.2 FV supplementation reduced tumor incidence in mice fed a high fat diet

**Figure 2:**
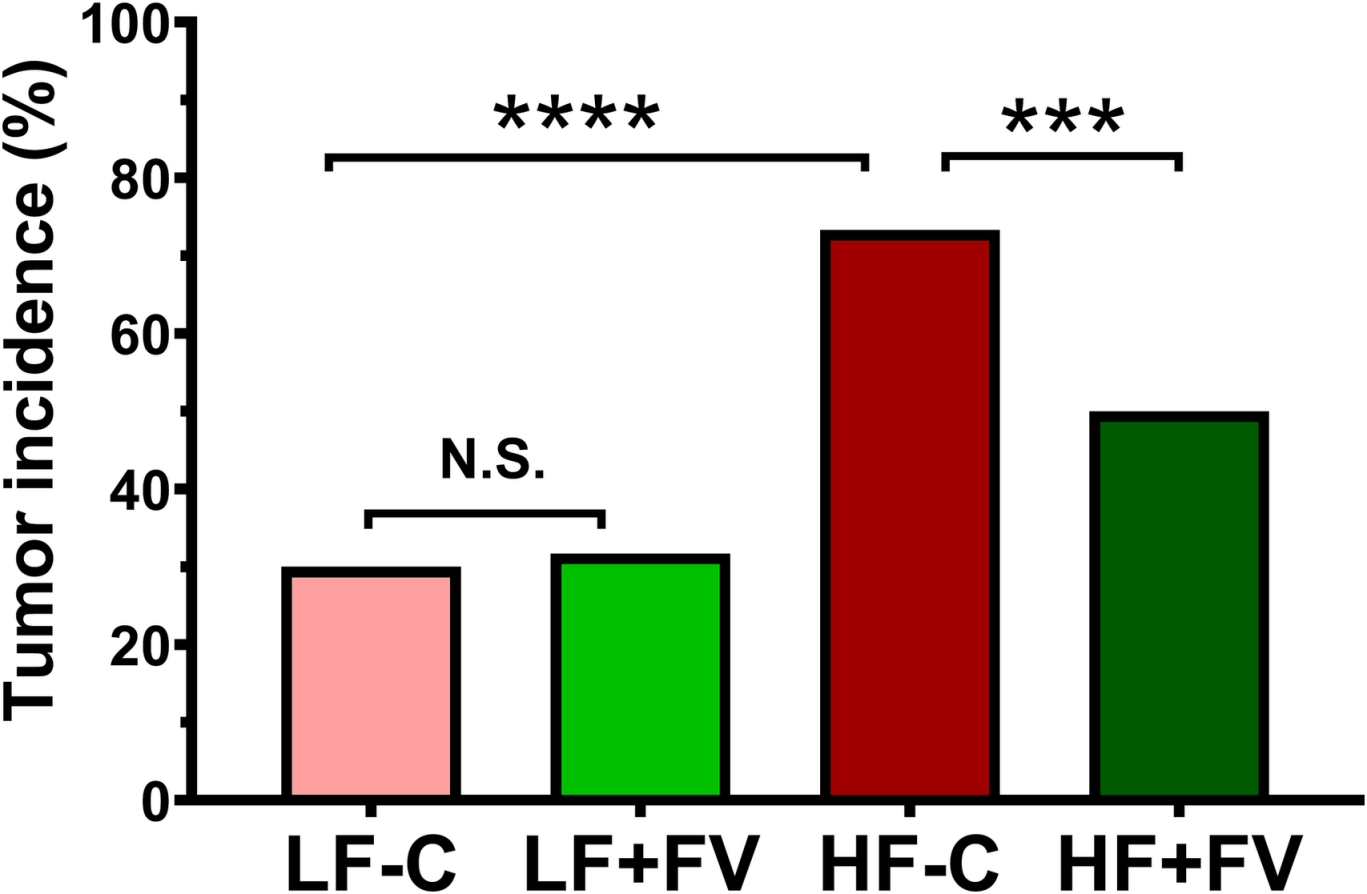
FV supplementation decreases tumor incidence in mice fed a high fat diet. Mice were fed LF-C (10% fat), HF-C (45% fat), or each diet supplemented with 15% FV (LF + FV, HF + FV) from when they were 5 weeks old until the first group reached 50% mortality, which was the HF-C group at 21 months. The total number of mice with tumor and the tumor type were recorded. A two-sided Fisher’s exact test was used for the comparison. Values are percentage of tumor incidence, *n* = 60, ^***^p < 0.01 and ^****^p < 0.001, N.S. not significant.

We observed spontaneous tumor formation among the mice that died before termination and the mice at termination in all diet groups, with some mice having more than one type of cancerous lesions (Tables 2, 3). The most prevalent type of tumor was liver tumor, followed by lung, intestinal, and seminal vesicle tumors. Overall tumor incidence was significantly higher in the HF-C group (73.3%) than in the LF-C group (30.0%, *p* < 0.0001). Mice fed the HF-FV diet had 23.3% lower tumor incidence compared to those fed the HF-C diet (*p* = 0.014), while there was no difference in tumor incidence between LF-C (30.0%) and the LF + FV groups (31.7%) (Figure 2).

**Table 2:** Death and tumor incidence in mice fed the different diets (*n* = 60/group).

	LF-C	LF + FV	HF-C	HF + FV
Mice dead before termination (*n*)	12	10	30	16
Mice with tumor before termination (*n*)	2	3	16	2
Mice terminated at 21 months (*n*)	48	50	30	44
Mice with tumor at termination (*n*)	16	16	28	28

**Table 3:** Tumor types in mice fed the 4 different diets (*n* = 60 mice/group).

Diet group	Total tumor (*n*)	Liver tumor (*n*)	Lung tumor (*n*)	Intestinal tumor (*n*)	Seminal vesicle tumor (*n*)
LF-C	18	12	5	1	3
LF + FV	19	15	5	2	1
HF-C	44	40	3	1	0
HF + FV	30	27	6	1	1

### 3.3 FV supplementation affected weight gain and body composition depending on age and health status

**Figure 3:**
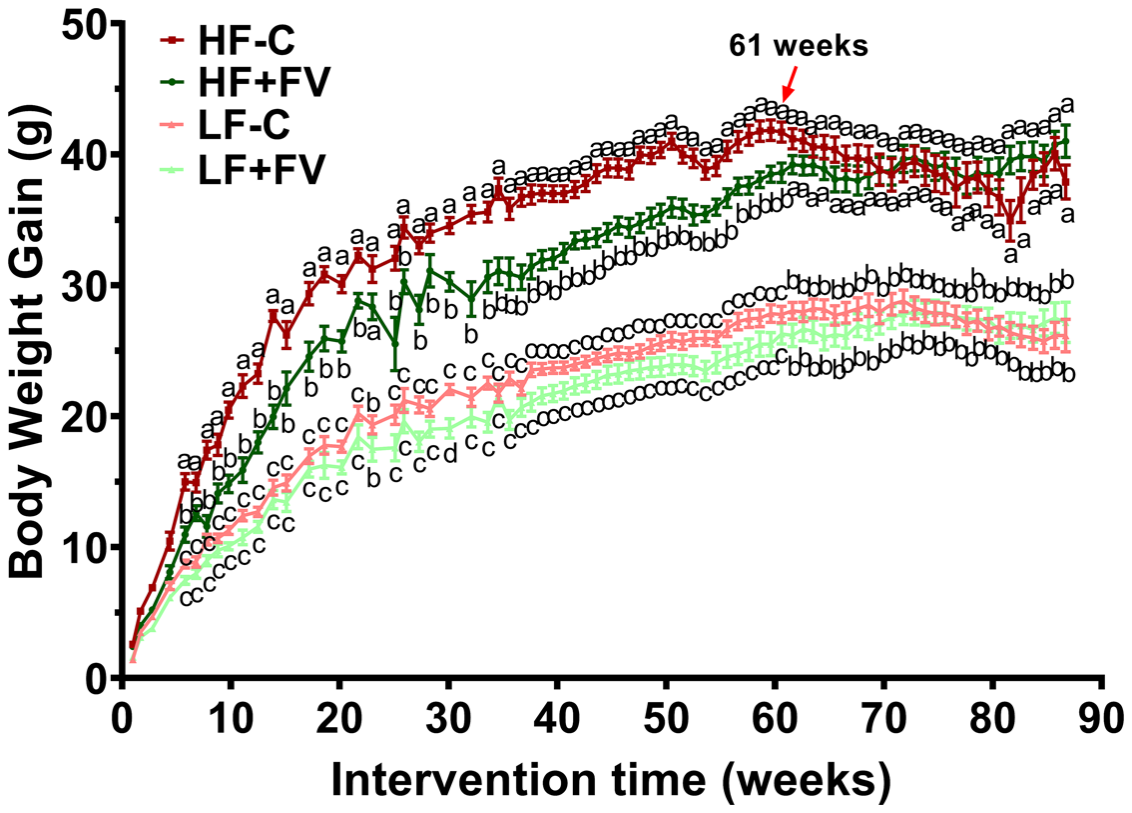
Effect of FV supplementation on body weight gain mice were fed LF-C (10% fat), HF-C (45% fat), or each diet supplemented with 15% FV (LF + FV, HF + FV) from when they were 5 weeks old until the first group reached 50% mortality, which was the HF group at 21 months. Body weight was recorded weekly. Means of body weight were compared across groups using one-way ANOVA, followed by Tukey’s *post hoc* test. Values are mean ± SEM, *n* = 30–60. Labeled means without a common letter significantly differ at *p* < 0.05.

Body weight in the HF-C group was significantly higher than that in the LF-C group at 90 weeks (21 months). Compared with the HF-C group, the HF + FV group had significantly lower high fat diet-induced weight gain. However, the initial FV-associated reduction of body weight gain disappeared after 62 weeks (14 months). No significant differences were found in weight gain between the LF-C group and the LF + FV group at any time point except 30 weeks, at which time LF + FV had significantly lower weight compared to the LF-C group (Figure 3).

Because most of the mice in the HF-C group developed cancerous lesions, which may impact weight gain, we divided mice into two groups based on the presence or absence of tumor at any time during the study (Supplementary Figures S1, S2). We found that the effect of FV supplementation on weight gain in mice who developed tumor disappeared after 52 weeks (12 months) (Supplementary Figure S1), which was much earlier than that in mice without tumor (60 weeks) (Supplementary Figure S2). The results from this analysis suggest that tumor may contribute to the diminished weight-reducing effect of FV in mice fed a high-fat diet. The presence or absence of tumors did not impact the effect of FV on weight gain in mice fed a low-fat diet.

We examined body composition at both 6 months and 16 months. A two-way ANOVA analysis demonstrated a significant diet effect (*p* < 0.0001) as well as age effect (*p* < 0.0001) on fat mass; no significant diet × age interaction for fat mass was observed. Significant diet (*p* < 0.0001), age (*p* < 0.0001), and diet × age interaction (*p* = 0.0181) effects were observed for lean body mass. As expected, at 16 months whole body weight and fat mass were higher while lean mass was lower compared to 6 months in all diet groups, albeit to different degrees. These observations are in agreement with previous aging studies that demonstrated an age-associated increase in adiposity concomitant with a decrease in lean body mass. As some mice died before the MRI measurement at 16 months, we performed paired comparison analysis and found similar results (Supplementary Figure S3).

**Figure 4:**
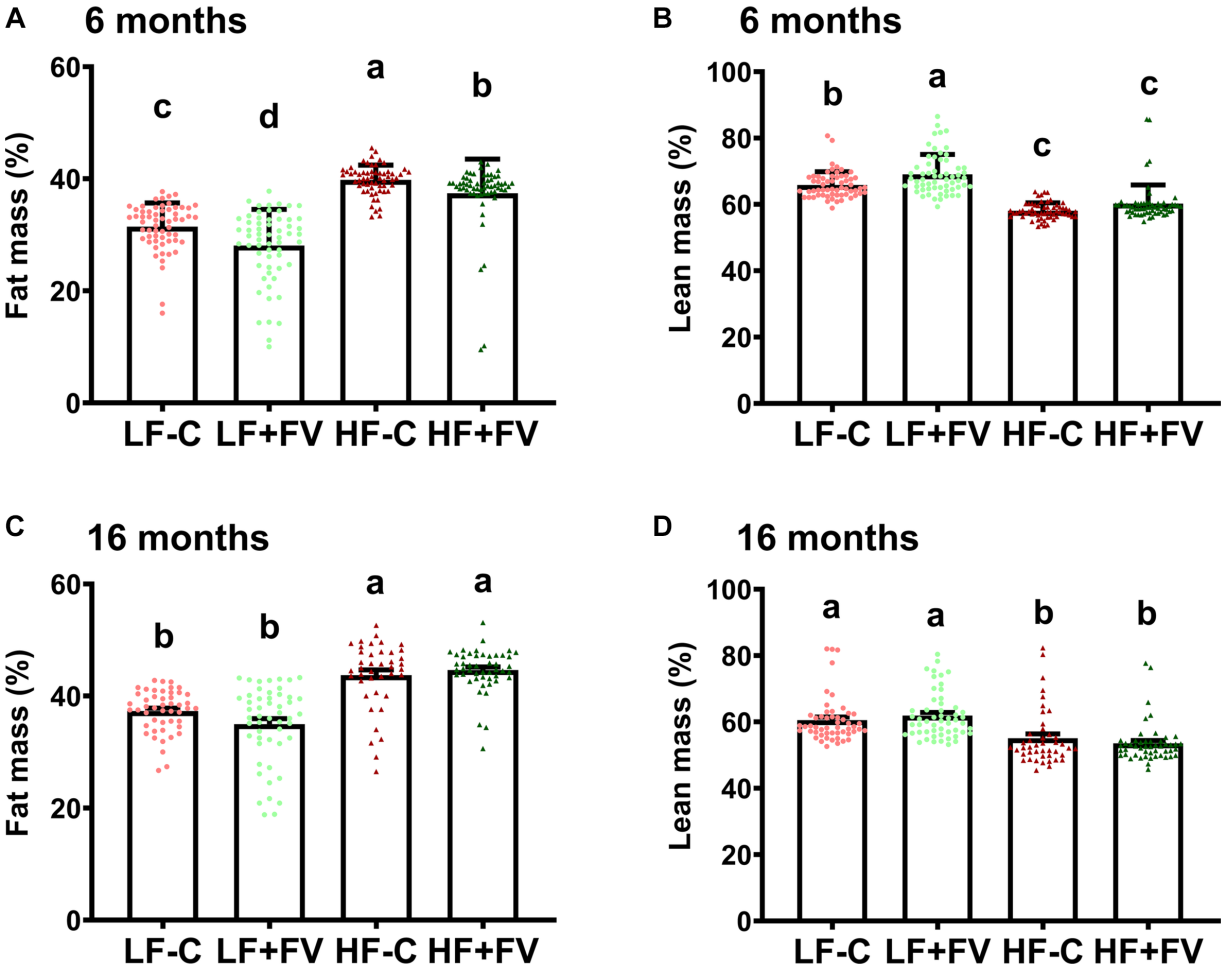
Effect of FV supplementation on body composition Mice (60/group) were fed LF-C (10% fat), HF-C (45% fat), or each diet supplemented with 15% FV (LF + FV, HF + FV) from when they were 5 weeks old until the first group reached 50% mortality, which was the HF-C group at 21 months. Body composition was assessed at 6 **(A,B)** and 16 months **(C,D)**, respectively. Means of fat mass and lean mass were compared across groups using one-way ANOVA, followed by Tukey’s *post hoc* test. Values are mean ± SEM, *n* = 60 at 6 months time point, and *n* = 41–55 at 16 months time point. Labeled means without a common letter significantly differ at *p* < 0.05.

At 6 months, the HF-C group had 26.5% more fat mass (*p* < 0.0001, *n* = 60) than the LF-C group. The HF + FV group had 6.4% less fat mass (*p* < 0.0001, *n* = 60) in comparison to the HF-C group, while LF + FV group had 12.0% less fat mass (*p* = 0.0019, *n* = 60) when compared to the LF-C group (Figures 4A,B). Conversely, the HF-C group had 13.5% less lean mass compared to the LF-C group (*p* < 0.0001, *n* = 60), and the HF + FV group trended towards having more lean mass (*p* = 0.0572, *n* = 60) compared to the HF-C group. The LF + FV group had 4.6% more lean tissue mass than that of the LF-C group (*p* < 0.0001, *n* = 60) (Figure 4B). These results suggest that FV supplementation for 6 months reduces fat mass in both mice fed LF-C and HF-C diet. At 16 months, the HF-C group maintained a significantly higher fat mass and lower lean mass than that of the LF-C group. The protective effect of FV supplementation on body composition was not observed at 16 months (64 weeks) (Figures 4C,D). The lack of FV effect at 16 months was not due to presence or absence of tumors (Supplementary Figure S4), although, at 6 months mice without tumor and fed the LF + FV had lower fat mass and higher lean mass compared to those fed the LF-C diet with tumor (Supplementary Figure S4). Spearman correlation analysis indicated that fat mass was positively correlated with tumor incidence (rho = 0.3157, *p* < 0.0001) and negatively associated with lean mass (rho = −0.3026, *p* < 0.0001).

### 3.4 FV supplementation improved diet- and age-related blood lipid changes

We performed blood lipid profile analysis at 12 and 21 months. In comparing the blood lipid levels including cholesterol, TG, LDL-c, HDL-c, and VLDL-c at 12 versus 21 months, two-way ANOVA analysis demonstrated a significant diet effect (*p* < 0.0001) and diet × age interaction (*p* < 0.0001). No age effect was observed except for the LDL-c/HDL-c ratio (*p* = 0.0005). Paired *t*-test analysis demonstrated that, in general, mice at older age (21 months) had significantly higher blood lipid levels in LF-C and LF + FV diet groups compared to their younger age (12 months) (Supplementary Figure S5). The blood lipid levels in HF-C group at older age, however, were significantly lower compared to their younger age. The differences in blood lipid parameters between older and younger age in mice fed HF + FV diet exhibited great variation. For example, blood TG, HDL-c, and VLDL-c were significantly lower at older age mice than at younger age and the ratio of LDL-c to HDL-c were remarkably higher at older age than at younger age, while there were no differences in blood cholesterol and LDL-c levels between the two life stages (older and younger age) (Supplementary Figure S5).

**Figure 5:**
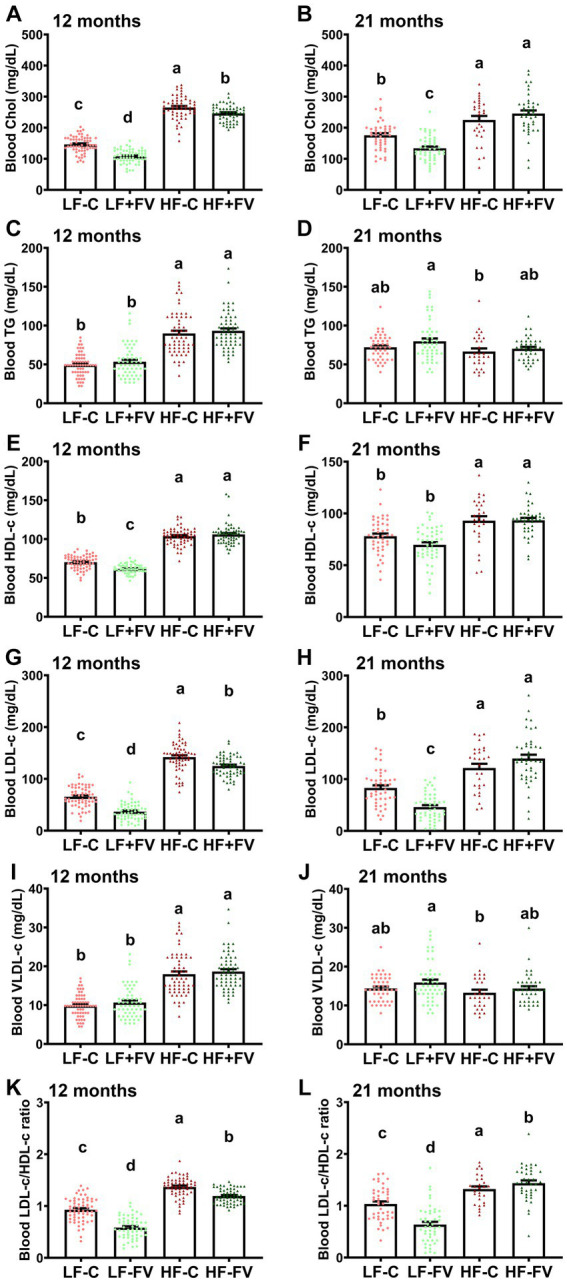
Effect of FV supplementation on blood lipid profile. Mice (60/group) were fed LF-C (10% fat), HF-C (45% fat), or each diet supplemented with 15% FV (LF + FV, HF + FV) from when they were 5 weeks old until the first group reached 50% mortality, which was the HF-C group at 21 months. Blood lipid profile was assessed at 12 months **(A–F)** and 21 months **(G–L)**. Means of blood cholesterol, TG, HDL-c, LDL-C, VLDL-c, and ratio of LDL-c/HDL-c were compared across groups using one-way ANOVA, followed by Tukey’s *post hoc* test. Values are mean ± SEM, *n* = 57–60 at 6 months time point, and *n* = 28–49 at 16 months time point. Labeled means without a common letter significantly differ at *p* < 0.05.

Blood lipid profile analysis showed that mice fed the HF-C diet had significantly higher blood cholesterol levels than those fed the LF-C diet at both 12 and 21 months. Both the LF + FV group and HF + FV group had lower blood cholesterol levels compared to their respective control mice at 12 months (Figure 5A). While the LF + FV group continued to exhibit lower blood cholesterol levels than the LF-C group at 21 months, no such difference was observed between the HF + FV group and HF-C group (Figure 5G) at 21 months. Similarly, the HF-C group had significantly higher blood LDL-c levels than the LF-C group, and both the LF + FV group and the HF + FV group had lower blood LDL-c levels compared to their respective control mice at 12 months (Figure 5D). At 21 months, the LF + FV group continued to exhibit lower blood cholesterol and LDL-c levels compared to the LF-C group. However, these differences between the HF-C and HF + FV group disappeared at 21 months (Figure 5J). FV supplementation did not have a significant effect on HDL-c level in mice fed a HF-C diet at either 12 or 21 months time points, nor in those fed the LF-C diet at 21 months time point. Although mice fed the LF + FV had significantly lower HDL-c level compared to those fed the LF-C at 12 months (Figure 5C), LF-FV mice had lower blood LDL-c/HDL-c ratio compared to their respective control mice at this time point, given their more pronounced effect on LDL-c levels. Similarly, HF-FV mice had lower blood LDL-c/HDL-c ratio compared to the respective control mice at 12 months (Figure 5F). We found that the LF + FV group continued to have lower blood LDL-c/HDL-c ratio than the LF-C group at 21 months. Unexpectedly, the HF + FV mice had higher blood LDL-c/HDL-c ratio than HF-C group (Figure 5L) at 21 months. FV supplementation did not affect blood levels of TG and VLDL-c (Figure 5).

Taken together, these results suggest that FV supplementation improves high fat diet-induced blood dyslipidemia up until middle age (12 months), however, this effect does not appear to last to older age (21 months). In contrast, the effect of FV supplementation on blood lipids is maintained in the mice fed LF-C diet throughout the lifespan.

Spearman correlation analysis showed that tumor incidence was positively correlated with blood cholesterol and LDL-c levels at both 12 months (rho = 0.3303, *p* < 0.0001, and rho = 0.3131, *p* < 0.0001, respectively) and 21 months (rho = 0.3238, *p* < 0.0001, and rho = 0.3571, *p* < 0.0001, respectively). Further, mouse survival days were negatively associated with blood cholesterol and LDL-c levels at 21 months (rho = −0.1642, *p* = 0.0117, and rho = −0.1948, *p* = 0.0026, respectively). No correlations were found between survival days and blood cholesterol and LDL-c levels at 12 months. These results suggest that HF-diet induced dyslipidemia may contribute to reduced lifespan/survival and that FV supplementation may increase median lifespan in part through maintenance of healthy circulating lipid profiles.

### 3.5 FV supplementation suppressed diet- and age-related chronic inflammation

It is postulated that chronic inflammation contributes to the aging process and cancer incidence (27–29). We examined circulating pro-inflammatory cytokine levels and found that overall FV supplementation had an anti-inflammatory effect as indicated by lower blood levels of inflammatory cytokines such as monocyte chemoattractant protein-1 (MCP-1), interleukin (IL)-6, TNFα, and keratinocyte chemoattractant/human growth-regulated oncogene (KC/GRO). However, this effect was not entirely uniform among all the markers and exhibited some variation depending on age and diet.

Two-way ANOVA analysis demonstrated a significant age effect for all of the blood pro-inflammatory cytokines (*p* < 0.0001 for IL-6, TNFα, MCP-1, and KC/GRO), diet effect (*p* = 0.0006 for IL-6, *p* < 0.0001 for TNFα, MCP-1, KC/GRO), and a diet × age interaction effect (*p* = 0.0006 for IL-6, *p* = 0.0142 for MCP-1, and *p* < 0.0001 for TNFα and KC/GRO, respectively). A significant age effect (*p* < 0.0001), diet effect (*p* < 0.0001), and diet and age interaction (*p* < 0.0001) were also observed for the anti-inflammatory cytokine IL-10.

**Figure 6:**
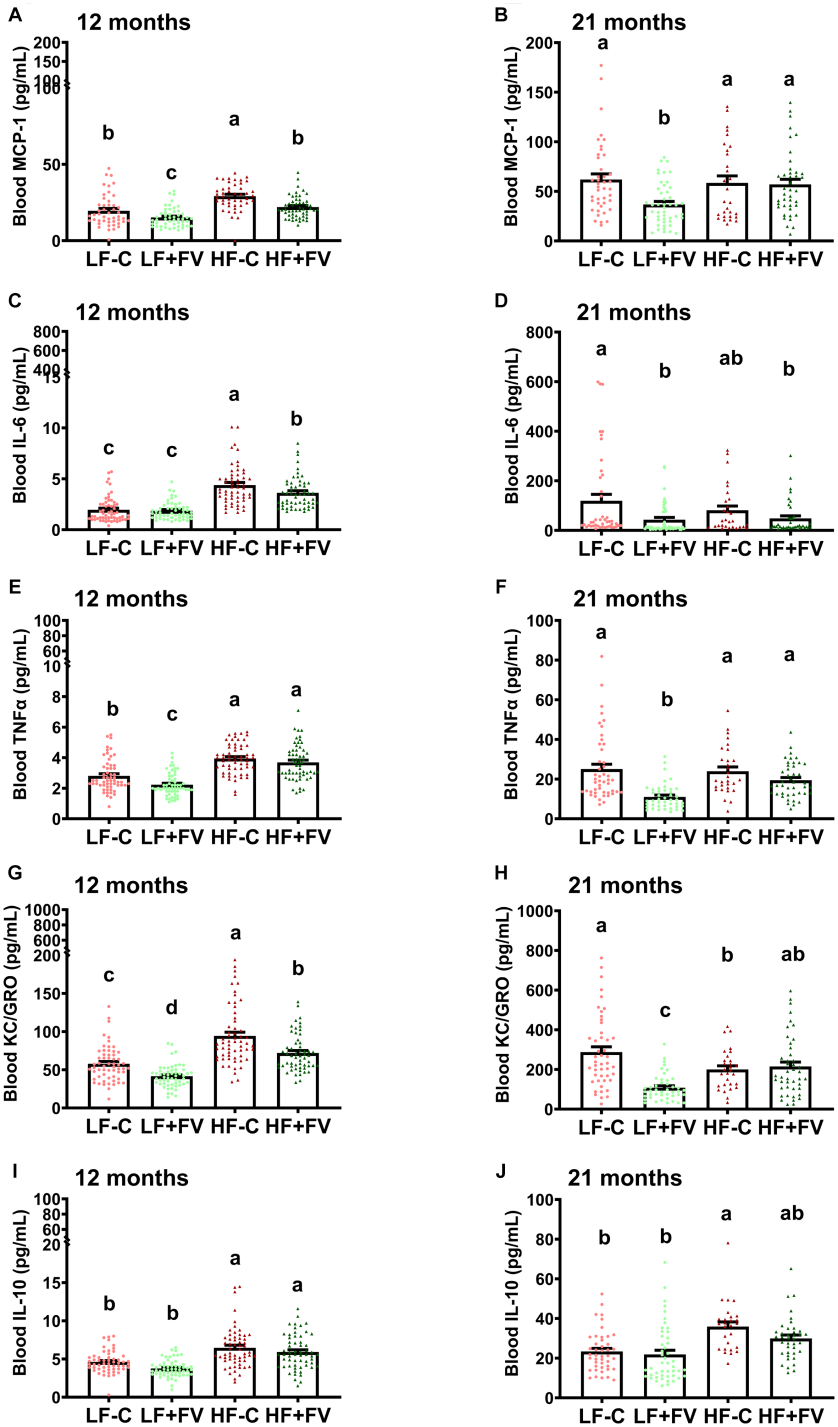
Effects of FV supplementation on levels of blood cytokines at 12 and 21 months mice (60/group) were fed LF-C (10% fat), HF-C (45% fat), or each diet supplemented with 15% FV (LF + FV, HF + FV) from when they were 5 weeks old until the first group reached 50% mortality, which was the HF-C group at 21 months. Blood cytokine levels were assessed at 12 months **(A–E)** and 21 months **(F–J)**, respectively. Means of blood cytokine levels were compared across groups using one-way ANOVA, followed by Tukey’s *post hoc* test. Values are mean ± SEM, *n* = 57–60 at 6 months time point, and *n* = 28–49 at 16 months time point. Labeled means without a common letter significantly differ at *p* < 0.05.

As expected, paired *t*-test analysis demonstrated that the level of inflammatory markers was significantly higher at 21 months in all diet groups compared to 12 months (Supplementary Figure S6). The level of anti-inflammatory cytokine IL-10 was also higher in all of the diet groups at 21 months in comparison to 12 months (Figure 6).

FV supplementation at 12 months significantly reduced circulating levels of some of the pro-inflammatory cytokine in mice fed the HF-C diet. This effect was not observed at 21 months.

At 12 months the HF-C group had significantly higher blood levels of pro-inflammatory chemokine MCP-1 than the LF-C group, and both LF + FV and the HF + FV groups had lower MCP-1 levels compared to their respective control mice (Figure 6A). At 21 months the LF + FV group continued to exhibit lower blood MCP-1 levels than the LF-C group while no such difference was found between the HF + FV group and the HF-C group (Figure 6F).

Additionally, the HF-C group had significantly higher blood levels of pro-inflammatory cytokine IL-6 than the LF-C group at 12 months. The HF + FV group had lower blood levels of IL-6 compared to the HF-C group. Unlike the observed difference in MCP-1, no difference in IL-6 was observed between the LF + FV group and the LF-C group (Figure 6B). However, the LF + FV group did exhibit lower blood levels of IL-6 than the LF-C group at 21 months, in contrast to the lack of such difference in HF-C mice at the same time point (Figure 6G).

At 12 months HF-C group had higher levels of TNFα in comparison to LF-C; however, by 21 months no significant difference in circulating TNFα levels was observed between LF-C and HF-C diets. Compared to the LF-C group, the LF + FV group had significantly lower blood TNFα levels at both 12 and 21 months, respectively, however, this effect of F&V was not observed in the HF-C mice (Figures 6C,H).

The HF-C group had significantly higher blood levels of pro-inflammatory cytokine KC/GRO than the LF-C group at 12 months. Both LF + FV and the HF + FV group had lower blood levels of KC/GRO compared to their respective control mice (Figure 6D). At 21 months, while this effect was still present in the LF-C mice, it no longer existed in HF-C mice (Figure 6I).

IL-10 level was higher in the HF-C group in comparison to the LF-C group at both 12 and 21 months (Figures 6E,J). There was no significant difference in IL-10 level at either 12 months or 21 months between the LF + FV group and the LF-C group or between the HF + FV group and the HF-C group (Figures 6E,J).

Spearman correlation analysis demonstrated that at 12 months, tumor incidence was positively correlated with blood MCP-1 (rho = 0.2075, *p* = 0.0035), IL-6 (rho = 0.2916, *p* < 0.0001), TNFα (rho = 0.2866, *p* < 0.0001), and KC/GRO (rho = 0.2233, *p* = 0.0006), while the survival days were negatively correlated with blood MCP-1 (rho = −0.2521, *p* = 0.0004), IL-6 (rho = −0.2419, *p* = 0.0003), TNFα (rho = −0.2651, *p* < 0.0001), and KC/GRO (rho = −0.2393, *p* = 0.0002). Furthermore, at 21 months tumor incidence was positively correlated with blood MCP-1 (rho = 0.1682, *p* = 0.0341) and TNFα (rho = 0.2388, *p* = 0.0021).

## 4 Discussion

Epidemiological evidence indicates that consumption of FV is negatively associated with the incidence of certain cancers and positively associated with life expectancy. However, the evidence supporting a causal relationship is lacking. In this prospective, pre-clinical study, we investigated the effect of life-long consumption of high levels of FV on median lifespan, key biological functions, and pathologies in normal weight and obese mice as well as the underlying mechanisms. We found that long-term FV supplementation extended median lifespan and reduced HF diet-associated mortality and tumor incidence in mice.

Further, our study demonstrated that FV supplementation significantly reduced high fat diet-induced weight gain in the initial 14 months. However, this FV-associated reduction of body weight gain disappeared after 14 months. When separating the mice into two groups based on the presence or absence of tumor at any time during the feeding period, we found that effect of FV supplementation on weight gain in mice who developed tumor disappeared after 52 weeks (12 months) of feeding, which was much earlier than that of mice without tumor (60 weeks). These results suggest that presence of tumor may contribute to the diminished weight-reducing effect of FV in mice fed a high fat diet. We also found that FV supplementation attenuated fat mass gain in mice fed either LF-C or HF-C diet at the early stage of the FV intervention (6 months of the feeding). However, this effect disappeared at 16 months. Spearman correlation analysis indicated that fat mass was positively correlated with tumor incidence in this animal model. Epidemiological evidence links FV consumption with reduced obesity and increased lifespan (30–32). Additionally, obesity is associated with the development of malignant tumors (33, 34). Therefore, our results suggest that, in the context of consuming a HF diet, FV supplementation may extend median lifespan and reduce tumor incidence at least partially through its beneficial effects on body weight gain and composition.

We found that FV supplementation improved high fat diet-induced blood dyslipidemia up until middle age (12 months), however, this effect did not appear to last to older age (21 months). The higher presence of tumors may have confounded the impact of FV on blood lipids at 21 months. In contrast, the effect of FV supplementation on blood lipids is maintained in the mice fed the LF-C diet throughout the lifespan. Furthermore, tumor incidence was positively correlated with blood cholesterol and LDL-c levels, and mouse survival days were negatively associated with blood cholesterol and LDL-c levels at 21 months. Given that obesity and dyslipidemia may be associated with increased cancer risk (35–37) and positive regulation of lipid metabolism might contribute to extended lifespan (38), our results suggest that HF-diet induced dyslipidemia may contribute to reduced lifespan/survival and that FV supplementation may increase median lifespan in part through maintaining a healthy circulating lipid profiles.

Aging is associated with increased inflammation, which in turn, has been noted to contribute to the age-associated diseases and pathologies. We demonstrated that middle age (12 months) mice fed the HF-C diet supplemented with FV supplementation had significantly lower levels of some of the pro-inflammatory cytokines compared to those fed a HF-C diet. Blood pro-inflammatory cytokines were positively correlated with tumor incidence and negatively correlated with the survival days. As chronic inflammation contributes to the aging process and cancer incidence (27–29), our results suggest that effects of FV supplementation on increased median lifespan and reduced tumor incidence are associated with, and might be partially mediated, by the suppression of pro-inflammatory cytokine production. We also found that circulating levels of anti-inflammatory cytokine IL-10 were substantially increased in both HF-diet fed groups (HF-C and HF-FV) in comparison to LF-diet fed groups (LF-C and LF-FV) at 12 months. The elevated blood IL-10 levels in animal fed a high fat diet has been observed by other investigators in high fat diet-induced obese mouse and rat model (39, 40), and this phenomenon may be a protective compensatory mechanism to counteract the pro-inflammatory and harmful metabolic effects of the high fat diet.

While the result of this first life-long prospective study on the effect of FV supplementation are significant in demonstrating the causal role of FV in reducing cancer and mortality and proposing potential underlying mechanisms, there are some limitations to the study. One of the limitations is that some of tumor vs. non-tumor comparisons were limited by the low number of animals in the HF-C group at 21 months. We did not anticipate this much higher mortality in this group. Future studies with a larger number of animals will be needed to conduct such sub-analysis. While epidemiological evidence supports the applicability of our findings to humans, prospective clinical trials will be needed to definitively confirm our findings in humans. Prospective human studies will albeit and to a large extent be limited to investigating the impact of F&V on key biological markers rather than disease and mortality. Our findings of significant correlations with lipids, inflammatory markers and BMI can guide selection of such markers. However, the precise mechanisms by which these biomarkers are associated with life span and cancer occurrence need further investigation.

In summary, these data show, for the first time, that life-long supplementation with high level of a variety of fruits and vegetables, causally extends median lifespan and reduces HF diet-associated mortality and tumor incidence in mice. These beneficial effects of FV are associated with, and may be mediated through, changes in key age- and obesity-associated biological markers, i.e., reduction of weight gain, improved blood lipid profile, and the reduced blood levels of pro-inflammatory cytokines. Further studies are needed to determine the relative contribution of each of the above to the observed effects of fruits and vegetables.

## Data availability statement

The original contributions presented in the study are included in the article/Supplementary material, further inquiries can be directed to the corresponding authors.

## Ethics statement

The animal study was approved by Institutional Animal Care and Use Committee of Tufts University. The study was conducted in accordance with the local legislation and institutional requirements.

## Author contributions

WG: Conceptualization, Data curation, Formal analysis, Investigation, Methodology, Project administration, Software, Supervision, Validation, Writing – original draft, Writing – review & editing. EO: Investigation, Writing – review & editing. DW: Conceptualization, Investigation, Writing – review & editing. LL: Investigation, Writing – review & editing. RB: Investigation, Writing – review & editing. SB: Data curation, Investigation, Writing – review & editing. SM: Conceptualization, Funding acquisition, Supervision, Writing – review & editing.

## References

[1] Kennedy BJ. Aging and cancer. Oncology. (2000) 14:1731–3. 11204375

[2] Macias RIR Monte MJ Serrano MA Gonzalez-Santiago JM Martin-Arribas I Simao AL. Impact of aging on primary liver cancer: epidemiology, pathogenesis and therapeutics. Aging. (2021) 13:23416–34. doi: 10.18632/aging.203620, PMID: 34633987 PMC8544321

[3] Pinti M Appay V Campisi J Frasca D Fulop T Sauce D. Aging of the immune system: focus on inflammation and vaccination. Eur J Immunol. (2016) 46:2286–301. doi: 10.1002/eji.201546178 27595500 PMC5156481

[4] Pereira B Xu XN Akbar AN. Targeting inflammation and immunosenescence to improve vaccine responses in the elderly. Front Immunol. (2020) 11:583019. doi: 10.3389/fimmu.2020.583019, PMID: 33178213 PMC7592394

[5] Jose SS Bendickova K Kepak T Krenova Z Fric J. Chronic inflammation in immune aging: role of pattern recognition receptor crosstalk with the telomere complex? Front Immunol. (2017) 8:1078. doi: 10.3389/fimmu.2017.01078, PMID: 28928745 PMC5591428

[6] Bazzano LA He J Ogden LG Loria CM Vupputuri S Myers L. Fruit and vegetable intake and risk of cardiovascular disease in US adults: the first National Health and Nutrition Examination Survey epidemiologic follow-up study. Am J Clin Nutr. (2002) 76:93–9. doi: 10.1093/ajcn/76.1.93, PMID: 12081821

[7] Johnsen SP Overvad K Stripp C Tjonneland A Husted SE Sorensen HT. Intake of fruit and vegetables and the risk of ischemic stroke in a cohort of Danish men and women. Am J Clin Nutr. (2003) 78:57–64. doi: 10.1093/ajcn/78.1.57, PMID: 12816771

[8] Joshipura KJ Ascherio A Manson JE Stampfer MJ Rimm EB Speizer FE. Fruit and vegetable intake in relation to risk of ischemic stroke. JAMA. (1999) 282:1233–9. doi: 10.1001/jama.282.13.1233 10517425

[9] Joshipura KJ Hu FB Manson JE Stampfer MJ Rimm EB Speizer FE. The effect of fruit and vegetable intake on risk for coronary heart disease. Ann Intern Med. (2001) 134:1106–14. doi: 10.7326/0003-4819-134-12-200106190-00010 11412050

[10] Trichopoulou A Costacou T Bamia C Trichopoulos D. Adherence to a Mediterranean diet and survival in a Greek population. N Engl J Med. (2003) 348:2599–608. doi: 10.1056/NEJMoa025039, PMID: 12826634

[11] Esmaillzadeh A Kimiagar M Mehrabi Y Azadbakht L Hu FB Willett WC. Fruit and vegetable intakes, C-reactive protein, and the metabolic syndrome. Am J Clin Nutr. (2006) 84:1489–97. doi: 10.1093/ajcn/84.6.1489 17158434

[12] Holt EM Steffen LM Moran A Basu S Steinberger J Ross JA. Fruit and vegetable consumption and its relation to markers of inflammation and oxidative stress in adolescents. J Am Diet Assoc. (2009) 109:414–21. doi: 10.1016/j.jada.2008.11.036 19248856 PMC2676354

[13] Wannamethee SG Lowe GD Rumley A Bruckdorfer KR Whincup PH. Associations of vitamin C status, fruit and vegetable intakes, and markers of inflammation and hemostasis. Am J Clin Nutr. (2006) 83:567–74. doi: 10.1093/ajcn.83.3.567 16522902

[14] Paul B Lewinska M Andersen JB. Lipid alterations in chronic liver disease and liver cancer. JHEP Rep. (2022) 4:100479. doi: 10.1016/j.jhepr.2022.100479, PMID: 35469167 PMC9034302

[15] Wang J Gao J Xu HL Qian Y Xie L Yu H. Citrus fruit intake and lung cancer risk: a meta-analysis of observational studies. Pharmacol Res. (2021) 166:105430. doi: 10.1016/j.phrs.2021.105430 33529754

[16] Guo XF Shao XF Li JM Li S Li KL Li D. Fruit and vegetable intake and liver cancer risk: a meta-analysis of prospective cohort studies. Food Funct. (2019) 10:4478–85. doi: 10.1039/C9FO00804G 31364650

[17] Kunzmann AT Coleman HG Huang WY Cantwell MM Kitahara CM Berndt SI. Fruit and vegetable intakes and risk of colorectal cancer and incident and recurrent adenomas in the PLCO cancer screening trial. Int J Cancer. (2016) 138:1851–61. doi: 10.1002/ijc.29922, PMID: 26559156 PMC6528653

[18] Guo WWD Li L Ortega E Liu Y Thomas M Nikolova-Karakashian M. Prevention of non-alcoholic fatty liver disease by fruits and vegetables supplementation in mice is associated with their antioxidant property. Curr Dev Nutr. (2020) 4:1523. doi: 10.1093/cdn/nzaa068_008

[19] Guo W Wu D Dao MC Li L Lewis ED Ortega EF. A novel combination of fruits and vegetables prevents diet-induced hepatic steatosis and metabolic dysfunction in mice. J Nutr. (2020) 150:2950–60. doi: 10.1093/jn/nxaa259, PMID: 32939550 PMC7919336

[20] Guo W Kim SH Wu D Li L Ortega EF Thomas M. Dietary fruit and vegetable supplementation suppresses diet-induced atherosclerosis in LDL receptor knockout mice. J Nutr. (2021) 151:902–10. doi: 10.1093/jn/nxaa410, PMID: 33561256

[21] Jin Y Cui X Singh UP Chumanevich AA Harmon B Cavicchia P. Systemic inflammatory load in humans is suppressed by consumption of two formulations of dried, encapsulated juice concentrate. Mol Nutr Food Res. (2010) 54:1506–14. doi: 10.1002/mnfr.200900579, PMID: 20425759

[22] Arvanitaki ES Stratigi K Garinis GA. DNA damage, inflammation and aging: insights from mice. Front Aging. (2022) 3:973781. doi: 10.3389/fragi.2022.973781, PMID: 36160606 PMC9490123

[23] Li S Kim HE. Implications of sphingolipids on aging and age-related diseases. Front Aging. (2021) 2:797320. doi: 10.3389/fragi.2021.797320 35822041 PMC9261390

[24] Lin BH Buzby JC Anekwe TD Bentley JT. U.S. food commodity consumption broken down by demographics, 1994–2008 U.S. Department of Agriculture ERS (2016).

[25] Shaner RL Allegood JC Park H Wang E Kelly S Haynes CA. Quantitative analysis of sphingolipids for lipidomics using triple quadrupole and quadrupole linear ion trap mass spectrometers. J Lipid Res. (2009) 50:1692–707. doi: 10.1194/jlr.D800051-JLR200, PMID: 19036716 PMC2724058

[26] Friedewald WT Levy RI Fredrickson DS. Estimation of the concentration of low-density lipoprotein cholesterol in plasma, without use of the preparative ultracentrifuge. Clin Chem. (1972) 18:499–502. doi: 10.1093/clinchem/18.6.499, PMID: 4337382

[27] Neves J Sousa-Victor P. Regulation of inflammation as an anti-aging intervention. FEBS J. (2020) 287:43–52. doi: 10.1111/febs.15061, PMID: 31529582

[28] Guerville F Bourdel-Marchasson I Dechanet-Merville J Pellegrin I Soubeyran P Appay V. Does inflammation contribute to cancer incidence and mortality during aging? A conceptual review. Cancers. (2022) 14:1622. doi: 10.3390/cancers14071622 35406394 PMC8996949

[29] Walker KA Basisty N Wilson DM 3rd Ferrucci L. Connecting aging biology and inflammation in the omics era. J Clin Invest. (2022) 132:e158448. doi: 10.1172/JCI158448, PMID: 35838044 PMC9282936

[30] Martel J Ojcius DM Ko YF Ke PY Wu CY Peng HH. Hormetic effects of phytochemicals on health and longevity. Trends Endocrinol Metab. (2019) 30:335–46. doi: 10.1016/j.tem.2019.04.001, PMID: 31060881

[31] Herpich C Muller-Werdan U Norman K. Role of plant-based diets in promoting health and longevity. Maturitas. (2022) 165:47–51. doi: 10.1016/j.maturitas.2022.07.003, PMID: 35914402

[32] Mello Rodrigues V Bray J Fernandes AC Luci Bernardo G Hartwell H Secchi Martinelli S. Vegetable consumption and factors associated with increased intake among college students: a scoping review of the last 10 years. Nutrients. (2019) 11:1634. doi: 10.3390/nu11071634 31319573 PMC6682864

[33] Gao L Yang T Xue Z Chan CKD. Hot spots and trends in the relationship between cancer and obesity: a systematic review and knowledge graph analysis. Life. (2023) 13:337. doi: 10.3390/life13020337 36836694 PMC9961916

[34] Sohn W Lee HW Lee S Lim JH Lee MW Park CH. Obesity and the risk of primary liver cancer: a systematic review and meta-analysis. Clin Mol Hepatol. (2021) 27:157–74. doi: 10.3350/cmh.2020.0176, PMID: 33238333 PMC7820201

[35] Shin HS Jun BG Yi SW. Impact of diabetes, obesity, and dyslipidemia on the risk of hepatocellular carcinoma in patients with chronic liver diseases. Clin Mol Hepatol. (2022) 28:773–89. doi: 10.3350/cmh.2021.0383 35934813 PMC9597232

[36] Neshat S Rezaei A Farid A Sarallah R Javanshir S Ahmadian S. The tangled web of dyslipidemia and cancer: is there any association? J Res Med Sci. (2022) 27:93. doi: 10.4103/jrms.jrms_267_22 36685020 PMC9854911

[37] Zhang D Xi Y Feng Y. Ovarian cancer risk in relation to blood lipid levels and hyperlipidemia: a systematic review and meta-analysis of observational epidemiologic studies. Eur J Cancer Prev. (2021) 30:161–70. doi: 10.1097/CEJ.0000000000000597, PMID: 32483012

[38] Johnson AA Stolzing A. The role of lipid metabolism in aging, lifespan regulation, and age-related disease. Aging Cell. (2019) 18:e13048. doi: 10.1111/acel.13048, PMID: 31560163 PMC6826135

[39] Alzahrani NS Alshammari GM El-Ansary A Yagoub AEA Amina M Saleh A. Anti-hyperlipidemia, hypoglycemic, and hepatoprotective impacts of pearl millet (*Pennisetum glaucum* L.) grains and their ethanol extract on rats fed a high-fat diet. Nutrients. (2022) 14:1791. doi: 10.3390/nu14091791 35565759 PMC9105973

[40] Lu X Dong Y Jian Z Li Q Gong L Tang L. Systematic investigation of the effects of long-term administration of a high-fat diet on drug transporters in the mouse liver, kidney and intestine. Curr Drug Metab. (2019) 20:742–55. doi: 10.2174/1389200220666190902125435, PMID: 31475894

[41] Guo W Wu D Li L Ortega E Smith D Meydani S. Long-term supplementation with fruits and vegetables prolongs lifespan and reduces tumor incidence in mice fed a western-style high-fat diet. Curr Dev Nutr. (2022) 6:22. doi: 10.1093/cdn/nzac047.022

