# Supplementary material for: Life-long consumption of high level of fruits and vegetables reduces tumor incidence and extends median lifespan in mice
Source: Front Nutr. 2023 Dec 6;10:1286792. doi: 10.3389/fnut.2023.1286792 (PMC10731956; doi:10.3389/fnut.2023.1286792)
Supplement: Supplementary file 1 [file Data_Sheet_1.PDF]

Supplementary Material

Life-long consumption of high level of fruits and vegetables reduces tumor incidence and extends median lifespan in mice

Weimin Guo [1,2]*, Edwin F. Ortega [1], Dayong Wu [1], Lijun Li [1], Roderick T. Bronson [3], Sarah K. Boehm[1], Simin N. Meydani [1]*

*** Correspondence:** Weimin Guo: wguo01@bu.edu
Simin Nikbin Meydani: simin.meydani@tufts.edu

1 Supplementary Figures and Tables

1.1 Supplementary Figures

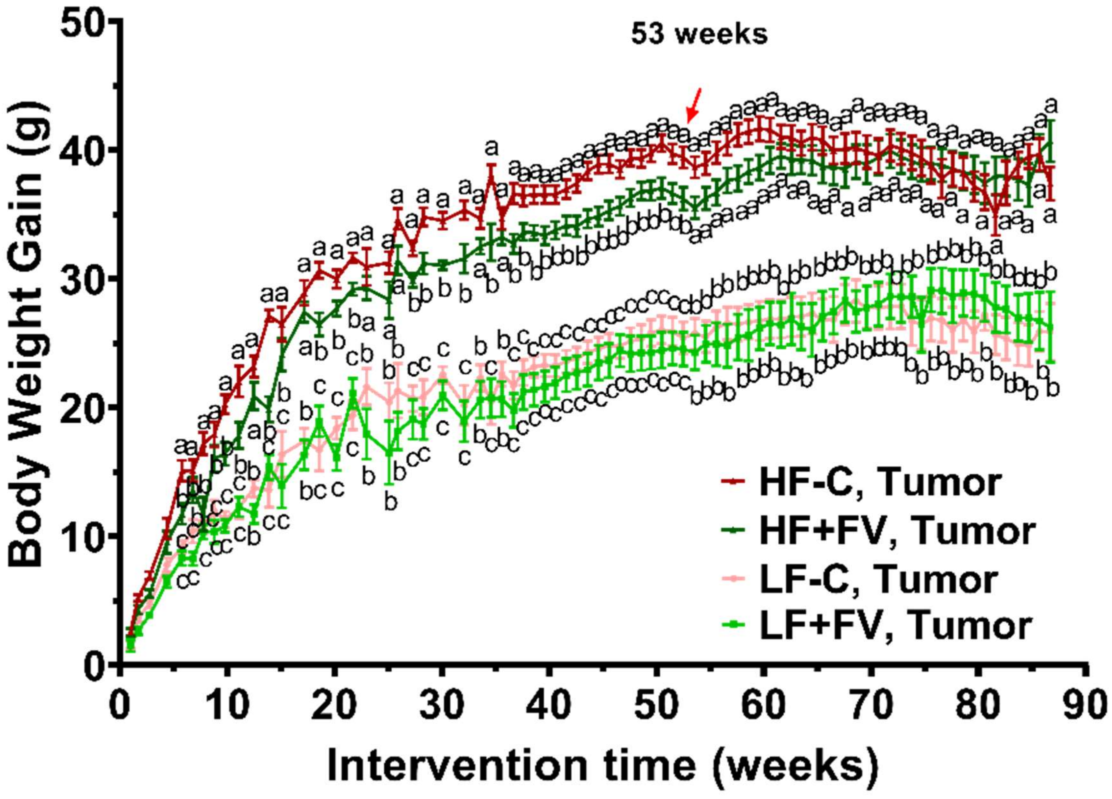

Supplementary Figure 1. Body weight gain in mice with cancer

Mice were fed LF-C (10% fat), HF-C (45% fat), or each diet supplemented with 15% FV (LF+FV, HF+FV) from when they were 5-week-old until the first group reached 50% mortality, which was the HF group at 21 months. Tumor incidence was recorded throughout the study. Mean body weight was compared across groups using one-way ANOVA, followed by Tukey's post hoc test. Values are mean ± SEM, n = 18 ~ 44. Labeled means without a common letter significantly differ at $p < 0.05$.

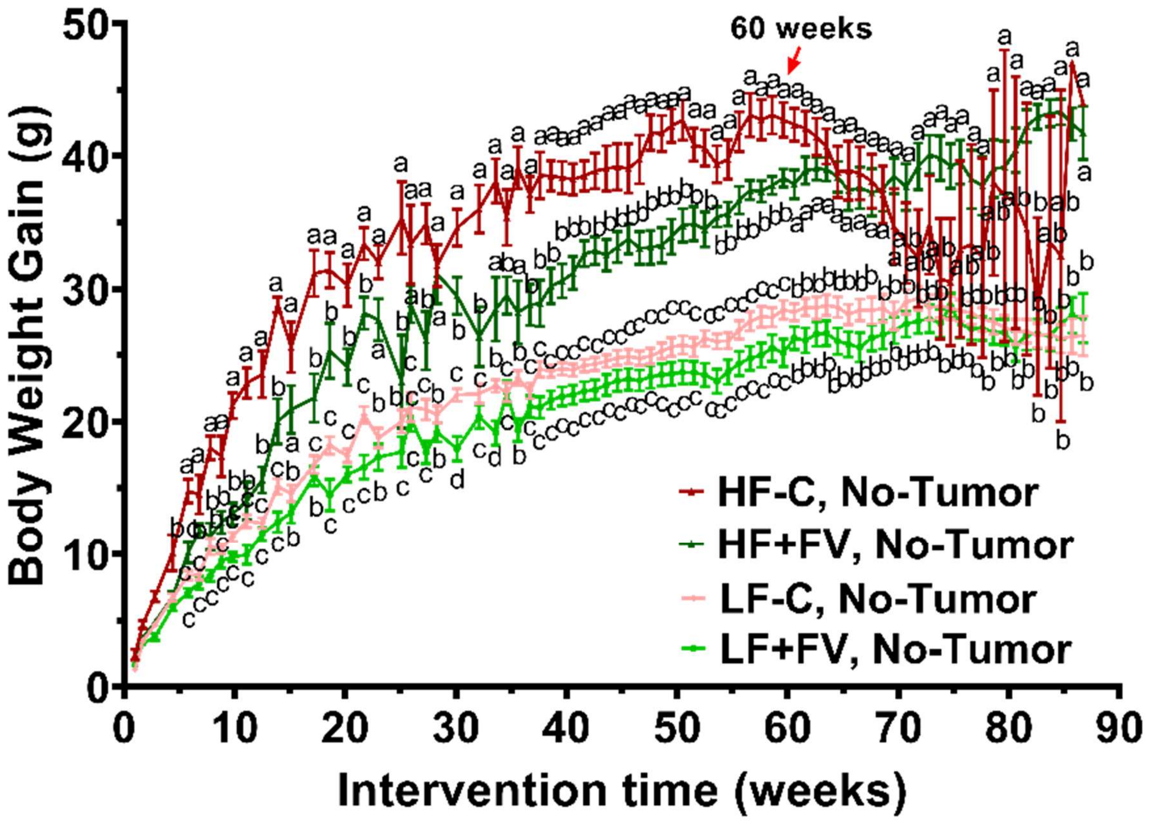

Supplementary Figure 2. Body weight gain in mice without cancer

Mice were fed LF-C (10% fat), HF-C (45% fat), or each diet supplemented with 15% FV (LF+FV, HF+FV) from 5-week-old until the first group reached 50% mortality, which was the HF group at 21 months. Means of body weight were compared across groups using one-way ANOVA, followed by Tukey's post hoc test. Values are mean ± SEM, n = 16 ~ 42. Labeled means without a common letter differ, $p < 0.05$.

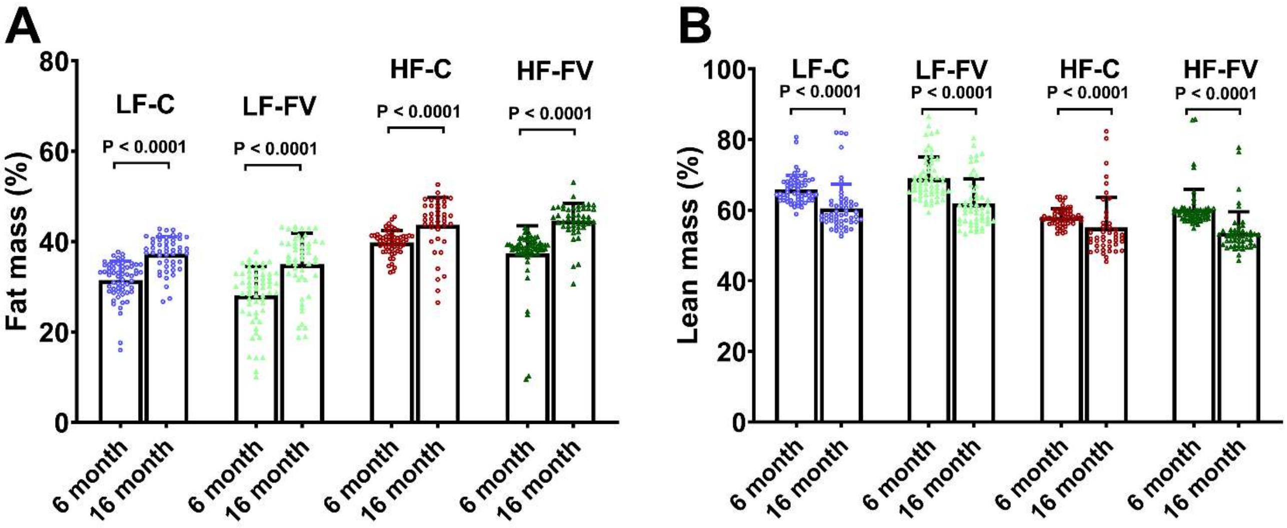

Supplementary Figure 3. Effect of FV supplementation on body composition and weight gain
Body weight and body composition were assessed at 6- and 16-months. Mean differences between two time-points within group was determined using two-tailed paired t test. Values are mean ± SEM, and n = 41 ~ 54.

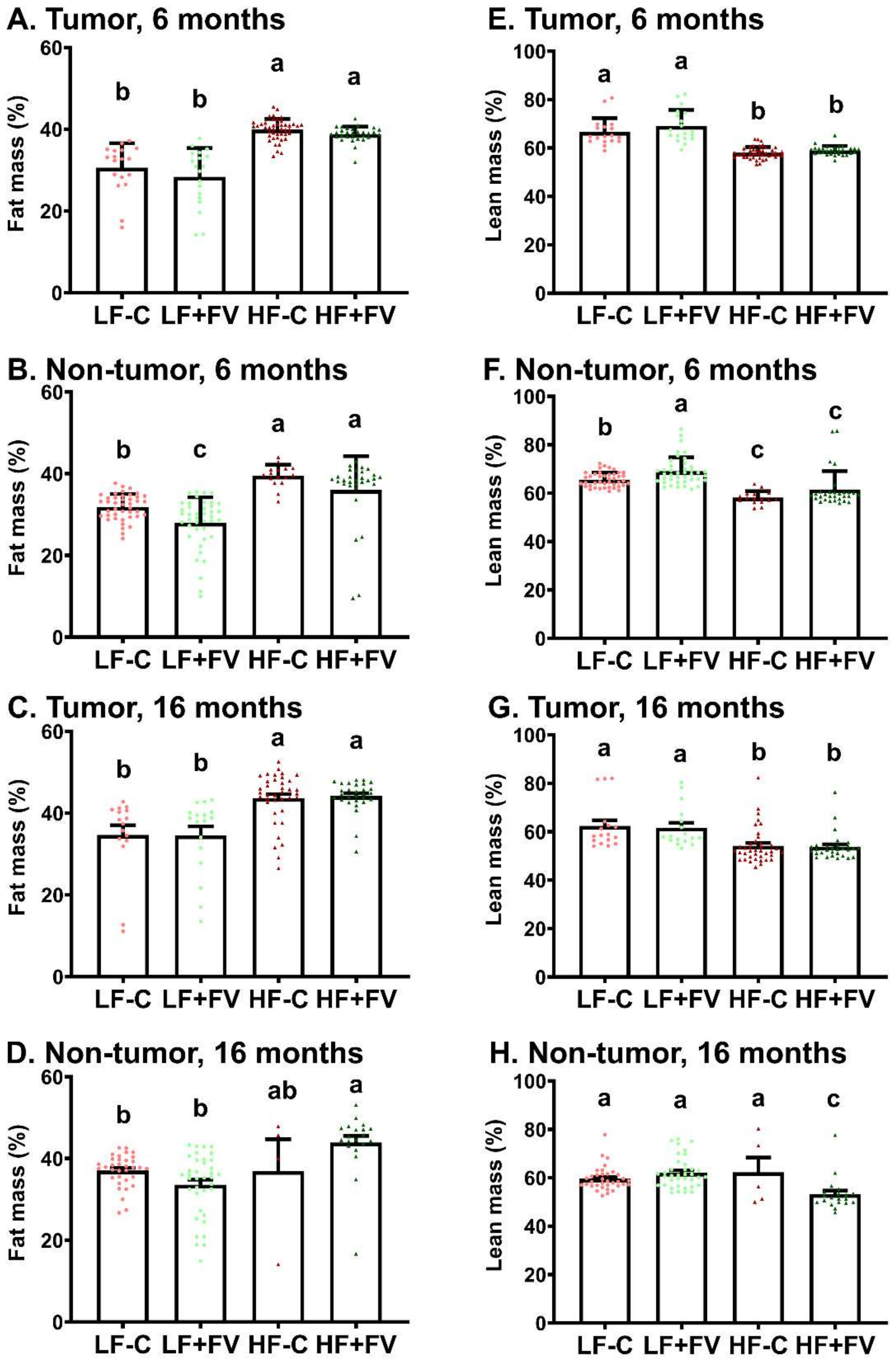

Supplementary Figure 4. Comparison of body composition and body weight assessed at 6 and 16 months in mice with or without tumor.

Body composition at 6 months (A–B and E–F) and 16 months (C–D and G–H) were analyzed in mice with or without tumor, respectively. Means of fat mass and lean mass were compared across groups using one-way ANOVA, followed by Tukey's post hoc test. Values are means ± SEM, n = 18 ~ 44 (tumor) or 16 ~ 42 (No-tumor) at 6-month time point, and n = 16 ~ 38 (tumor) or n = 4 ~ 37 (No-tumor) at 16-month time point. Labeled means without a common letter significantly differ, $p < 0.05$.

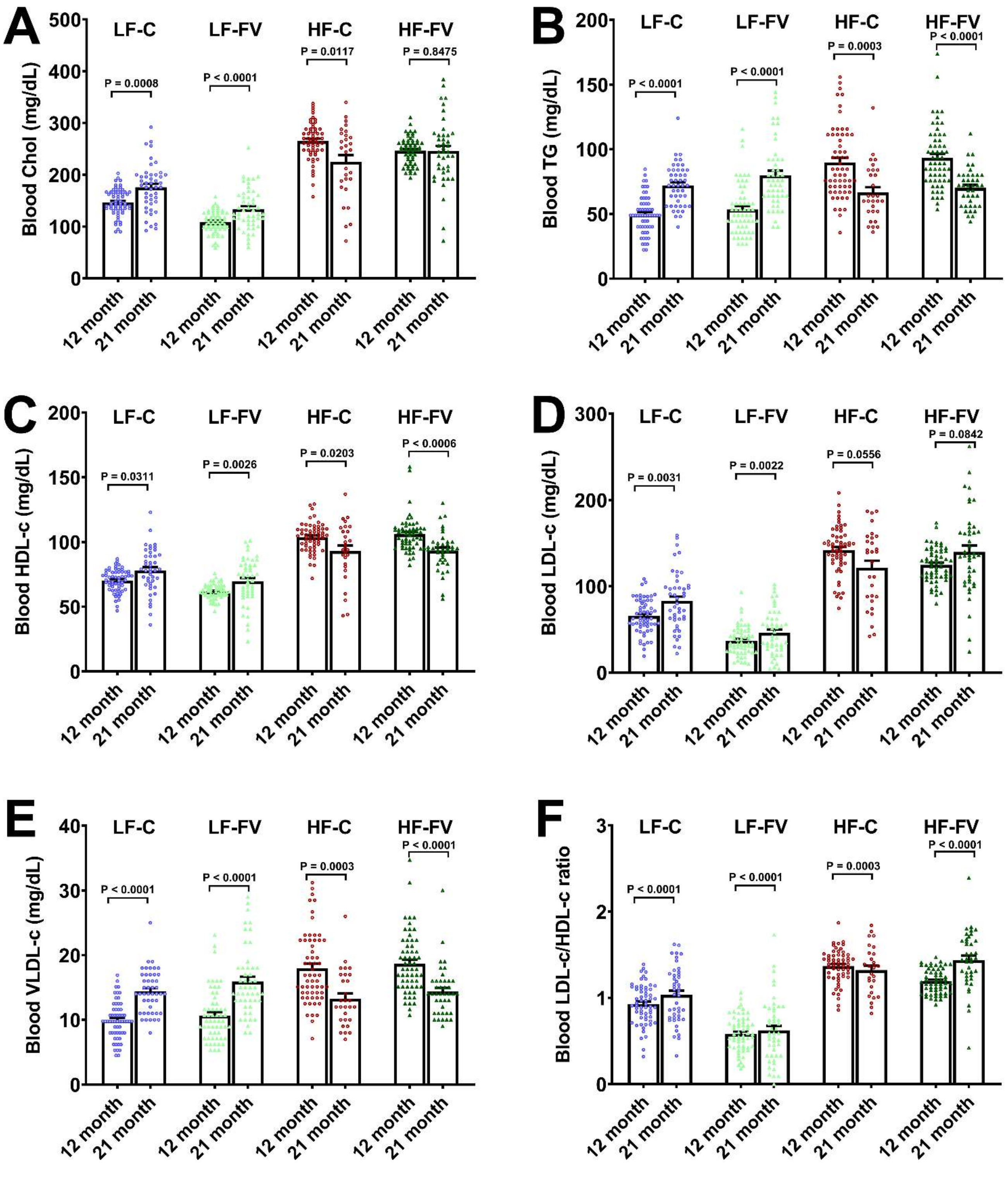

Supplementary Figure 5. Comparison of FV effect on blood lipid profile at 12 months and 21 months.

Mean differences of blood cholesterol, TG, HDL-c, LDL-C, and VLDL-c within diet group were compared using two-tailed paired t test. Values are mean ± SEM, n = 29 ~ 49.

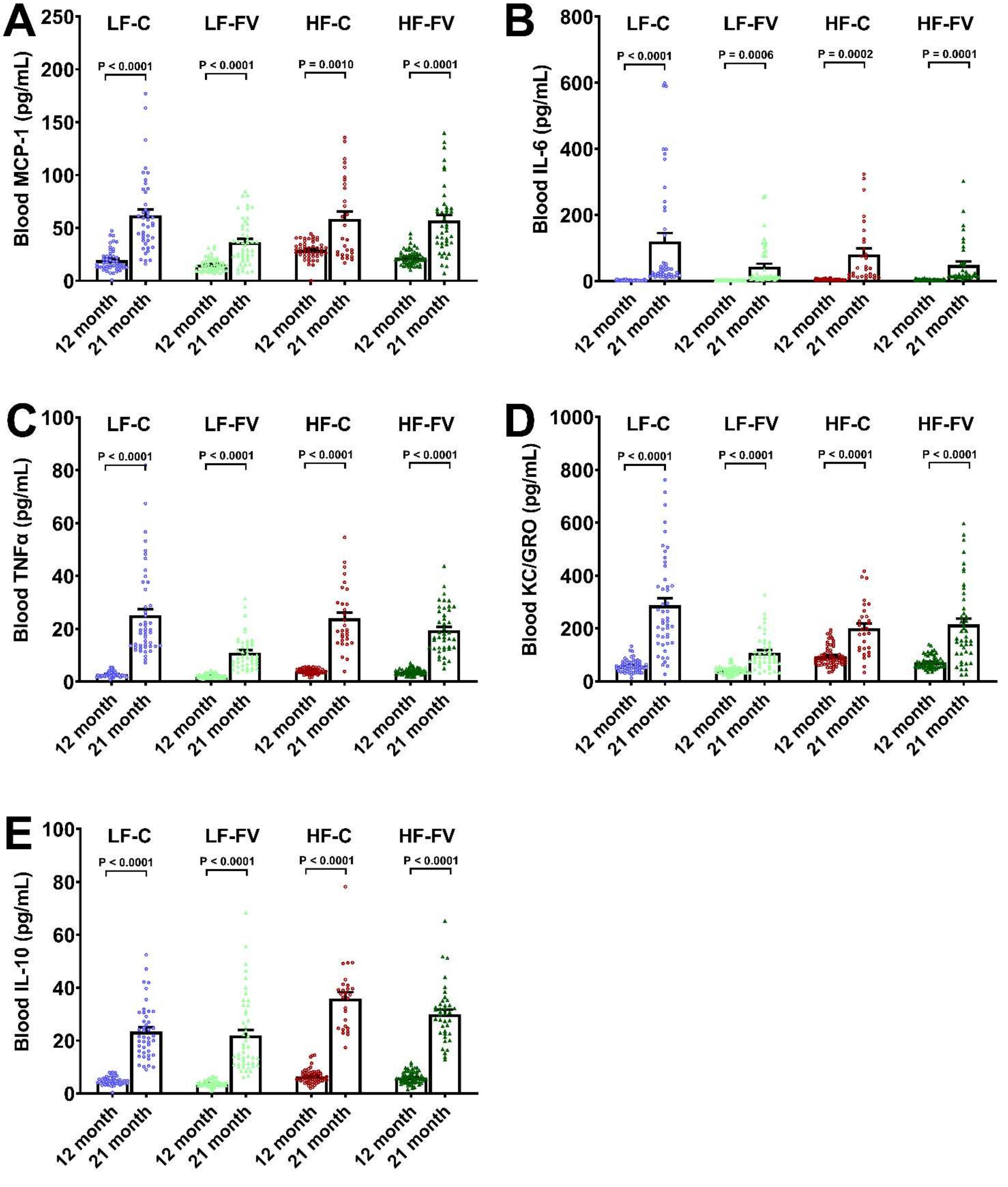

Supplementary Figure 6. Comparison of FV effect on blood cytokine levels at 12 months and 21 months.

Mean differences of blood levels of MCP-1, IL-6, TNFα, KC/GRO, and IL-10 within diet group were compared using two-tailed paired t-test. Values are mean ± SEM, n = 29 ~ 47.
